# Can systemic antibiotics reduce the need for surgical intervention in treating peri-implantitis?

**DOI:** 10.1038/s41432-024-01071-x

**Published:** 2024-10-09

**Authors:** Nidhi Parmar

**Affiliations:** MClinDent Prosthodontics Specialty Trainee, Eastman Dental Institute, London, United Kingdom

**Keywords:** Peri-implantitis, Dental implants, Dental implants, Antibiotics in dentistry, Antibiotic prophylaxis in dentistry

## Abstract

**Design:**

A retrospective cohort design was used to assess the long-term clinical effectiveness of systemic amoxicillin and metronidazole, used adjunctively with non-surgical peri-implantitis treatment (NST) and whether it prevents the need for further surgical interventions.

**Cohort selection:**

Of the 57 peri-implantitis patients examined, 45 patients were included in this study. The participants were divided into two subgroups in accordance to who had received NST with or without systemic antibiotics. Selection was based on prior participation in a three-month randomised control trial, ensuring comparability of data regarding peri-implantitis severity and treatment history.

**Data analysis:**

Data were extracted pre-treatment, 3-months post-treatment and at a long-term follow-up interval of 36 months. The primary outcome was the need for additional surgical intervention and was analysed via Kaplan–Meier analysis and Cox regression. A multitude of secondary clinical outcomes were evaluated using parametric and non-parametric tests, including peri- implant probing depth, bleeding scores and treatment success.

**Results:**

Overall, 62.2% of the 45 NST patients did not need surgical peri-implantitis treatment: 73.9% of the subgroup with antibiotics and 50% of the subgroup without antibiotics respectively. However, the difference between the two groups was not statistically significant (log-rank test, *p* = 0.110). The Cox regression analysis also displayed no significance over the first three years post-treatment (*p* = 0.115). Additionally, the study found that deeper peri- implant pockets at baseline significantly predicted the need for future surgical treatment (*p* = 0.031), highlighting the importance of initial disease severity in treatment outcomes.

**Conclusions:**

The study concludes that the adjunctive use of systemic amoxicillin and metronidazole with NST may delay but not statistically reduce or prevent a future surgical need. Although a short-term reduction in clinical inflammatory parameters was evident, the long-term effectiveness in altering the progression of peri-implantitis remains limited.

## A Commentary on

**Hakkers J, Vangsted T E, van Winkelhoff A J, de Waal Y C M**.

Do systemic amoxicillin and metronidazole during the non-surgical peri-implantitis treatment phase prevent the need for future surgical treatment? A retrospective long-term cohort study. *J Clin Periodontol* 2024; **51:** 997–1004.

**GRADE Rating:** 

## Commentary

Peri-implantitis is a pathological, inflammatory disease demarcated by mucosal inflammation and progressive bone loss around an osseointegrated dental implant. The aim of both NST and surgical treatment is to eliminate the pathogenic bacteria that colonise the implant surface^1^. The literature stipulates that although NST improves clinical parameters such as bleeding scores and probing depths, it often results in incomplete disease resolution^2^. This prompts for the exploration of adjunctive therapies such as systemic antibiotics to enhance NST, and potentially reduce the need for invasive surgical procedures.

This cohort study assesses the clinical efficacy of systemic amoxicillin and metronidazole as an adjunct to NSI. The study is of relevance as it correlates the need for surgical treatment after NSI, which does not exist in the current body of literature^3^. The authors express their prediction of no significant difference in surgical outcomes between patients treated with or without antibiotics, which is in line with the current literature^4^.

Overall, this paper follows the relevant CASP criteria for cohort studies. The retrospective nature of the study allows for observation over an extended period of 36 months. However, it introduces potential biases related to data collection and reliance on historical medical records. The study cohort was a convenience sample yielded from a previous randomised control trial, which introduces selection and information biases and limits data extrapolation to a larger population facing similar treatments. Methods and criteria for assessing the outcomes were standardised across participants. The results were clearly delineated and the primary outcomes were objectively measured. However, the secondary clinical outcomes had the potential for subjectivity and there was no indication of the use of blinding for outcome assessors.

The authors accounted for confounders by employing relevant statistical analyses and adjustments. For example, the adjusted Cox regression model and univariable and multivariable regression analyses scrutinised how the impact of antibiotics was influenced by other variables over time. However, it’s unclear if all relevant confounding factors, such as patient adherence to oral hygiene practices or other systemic health issues, were considered.

Other limitations affecting this study include the relatively small sample size, which reduced the statistical power to detect significant differences. Furthermore, the variability in each patient’s follow-up over the 36-months, introduces discrepancies in treatment outcome assessments. Along with collecting clinical data, future studies should also include radiographic findings at each interval to fully assess NST and surgical treatment success.

In summary, NSI with or without systemic amoxicillin and metronidazole does not significantly prevent peri-implantitis surgery. Despite the suggestive findings of this study, the data should be interpreted cautiously. Further higher quality studies with larger sample sizes are required to firmly establish this relationship.

Practice points

- The study stipulates that systemic antibiotics should not be routinely prescribed with NST to prevent future surgeries in peri-implantitis cases, in line with the principles of antibiotic stewardship.
- This study implies the decision of its benefit with NSI should be based on the severity of the initial condition.
